# Case Report: Anti-mGluR5 antibody-negative Ophelia syndrome with failed lymph node biopsy due to steroid therapy

**DOI:** 10.3389/fimmu.2023.1188154

**Published:** 2023-12-08

**Authors:** Yui Sanpei, Masahito Miura, Homare Funasaka, Akira Hanazono, Sachiko Kamada, Masashiro Sugawara

**Affiliations:** ^1^ Department of Neurology, Akita University Graduate School of Medicine, Akita, Japan; ^2^ Department of Molecular and Tumour Pathology, Akita University Graduate School of Medicine, Akita, Japan

**Keywords:** Ophelia syndrome, paraneoplastic limbic encephalitis, Hodgkin lymphoma, anti-mGluR5 antibody, steroid

## Abstract

Ophelia syndrome is paraneoplastic limbic encephalitis (PLE) with Hodgkin lymphoma. Some Ophelia syndrome patients have been reported as testing positive for anti-metabotropic glutamate receptor 5 (mGluR5) antibodies. However, we experienced a case of anti-mGluR5 antibody-negative Ophelia syndrome. The type of onset, neurological symptoms, and imaging as well as electroencephalographic findings were like previous reports except for a normal cell count in cerebrospinal fluid (CSF). Unfortunately, a lymph node biopsy failed and could not diagnose the patient before death because steroid treatment for limbic encephalitis had shrunk lymph nodes. We believe it is essential to accumulate cases of this syndrome and clarify the association between PLE and Hodgkin lymphoma so chemotherapy can be initiated even if malignant lymphoma cannot be pathologically proven or when antibodies cannot be measured or are negative.

## 1 Introduction

Ophelia syndrome is known as PLE in a patient with Hodgkin lymphoma and was first described by Carr (1) in his teenage daughter. Various neuropsychiatric abnormalities range from mood and personality changes to involuntary movements, headaches, disorientation, and amnesia.

When we see limbic encephalitis, steroids are often used early on, assuming autoimmune encephalitis that can be treated with immunotherapy, such as anti-voltage–gated potassium channel (VGKC) antibody-related encephalitis. However, in the case of Ophelia syndrome, steroids can shrink lymph nodes, and there is a risk of not obtaining the malignant lymphoma tissue necessary for diagnosis.

Anti-mGluR5 antibodies are known to be relevant for Ophelia syndrome. Some cases have been reported positive for anti-mGluR5 antibodies and have been reported as encephalitis with mGluR5 antibodies (2–6). In addition, anti-mGluR5 antibodies have recently been experimentally demonstrated to cause mice to induce behavioral changes, memory deficits, and anxiety after cerebrospinal fluid infusion of patients’ IgG (7). However, few institutions can evaluate the presence of antibodies at the start of treatment. In addition, antibodies have generally been undetectable in most paraneoplastic syndromes associated with malignant lymphoma (8). Knowing the association between PLE and Hodgkin lymphoma may reduce cases that cannot be diagnosed before death and improve patients’ prognoses. Our case of Ophelia syndrome presented with abnormal behavior, memory loss, impaired consciousness, and status epilepticus. Although malignant lymphoma was strongly suspected, steroid treatment for limbic encephalitis made antemortem diagnosis difficult. We report the clinical features of a patient with Ophelia syndrome who was negative for anti-mGluR5 antibodies in CSF and serum obtained before steroid therapy.

## 2 Case presentation

**Figure 1:**
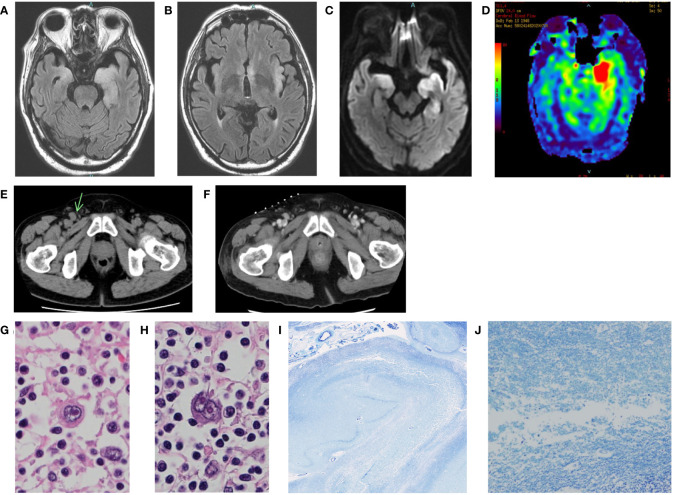
**(A-D)**: Brain MRI of the patient at the onset. **(A, B)** FLAIR. **(C)** DWI. **(D)** arterial spin labeling (ASL). Bilateral medial temporal lobes and left insular gyrus were swollen with high signal **(A-C)**. ASL images showed left dominant asymmetric hippocampus cerebral blood flow increase **(D)**. **(E)** Pelvic CT of the patient before IVMP. Mild lymph node enlargement in the inguinal regions. The right inguinal lymph node was the largest (arrow). **(F)** Pelvic CT of the patient after IVMP. The lymph node had shrunk. The hyperdense dots on the skin are markers placed for biopsy. **(G, H)** Histological findings of hematoxylin-eosin staining in the abdominal periaortic lymph node. Enlarged atypical cells proliferated, and Hodgkin and Reed-Sternberg cells were observed. **(I, J)** Histological findings of Klüver-Barrera staining in the left hippocampus. Spongiosis and neuronal loss were observed.

A man in his 70s with a 7-year history of erythroderma presented with amnesia. Fatigue, anorexia, and dyspnea preceded the neurological symptoms. Three days later, he developed a fever and mild altered mental status with disorganized speech and wandering. An MRI of the head showed abnormal signals in the bilateral medial temporal lobes and left insular gyrus (**Figures 1A-D**), leading to the suspicion of limbic encephalitis. He was referred to our hospital. Neurological examination revealed impaired consciousness (Glasgow Coma Scale was 8, E2V2M4) and left-hand automatism. The physical study showed no abnormal findings in the thorax and abdomen and no superficial lymph nodes swelling.

He developed status epilepticus and was intubated and ventilated under intravenous sedation. The CSF examination showed a normal cell count and a mildly elevated protein level (48 mg/dl). The oligoclonal band and IgG index were not tested. CSF cytology revealed only a small number of lymphocytes and no malignant cells. CSF flow cytometry could not be performed due to the small number of cells. Electroencephalography revealed diffuse slowing and periodic lateralized sharp waves in the left frontal lobe, but no extreme delta brush pattern was found. We suspected autoimmune encephalitis such as anti-VGKC antibody-associated encephalitis and paraneoplastic limbic encephalitis. As he developed status epilepticus requiring intensive care, we started intravenous methylprednisolone (IVMP; 1,000 mg/day for five days), considering the importance of early suppression of autoimmune inflammation from an early stage. We used acyclovir until a CSF-polymerase chain of herpes simplex virus proved negative.

Because soluble IL-2 receptor levels were high (4,457 IU/mL), we performed whole-body cancer screening for PLE, and CT scans revealed mild lymph node enlargement in the left axilla, mediastinum, abdominal periaortic region, and both inguinal regions (**Figure 1E**). His condition precluded a PET scan. Considering the possibility of PLE associated with malignant lymphoma, a biopsy was attempted from the right inguinal lymph node, which was the largest on CT images. However, the lymph node had shrunk under the influence of steroids (**Figure 1F**), and no helpful tissue could be obtained for diagnosis. Subsequently, another biopsy of the lymph node was attempted but failed. A biopsy of erythema on the left thigh also did not reveal findings suggesting malignant lymphoma. Because acute renal failure and thrombocytopenia appeared, we considered the possibility of intravascular malignant lymphoma and performed bone marrow and random skin biopsies but could not prove the presence of malignant lymphoma.

**Figure 2:**
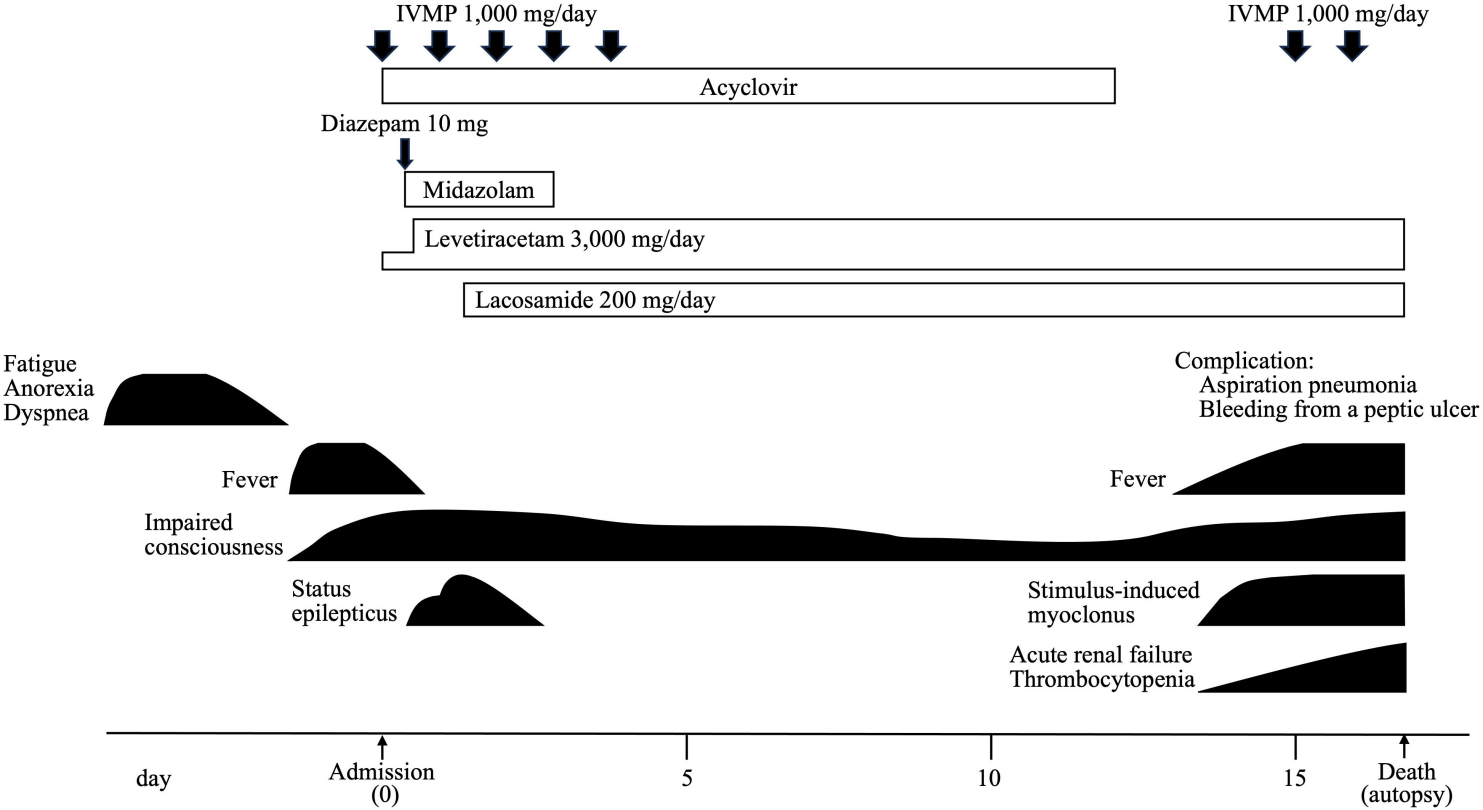
Clinical course of the patient.

IVMP temporarily improved his level of consciousness, and his seizures were controlled with levetiracetam and lacosamide alone. However, 15 days after the onset, his level of consciousness deteriorated again, and he developed stimulus-induced myoclonus. The patient died of aspiration pneumonia and bleeding from a peptic ulcer on the 17th day without treatment for malignant lymphoma. The clinical course of this case is shown in **Figure 2**.

We obtained the family’s consent and performed an autopsy. The lymph nodes showed various fibrosing processes, from capsular fibrosis to replacement with a fibrotic scar. Some nodes had a nodular growth pattern, with nodules surrounded by collagen bands. Scattered atypical large cells, e.g., Hodgkin, mummified, lacunar, and Reed-Sternberg cells were observed in the fibrosing lymph nodes (**Figures 1G, H**). Immunohistochemically, the atypical cells were positive for CD 30 and CD 15 and negative for CD 3 and CD 20. The diagnosis of nodular sclerosis classical Hodgkin’s lymphoma was confirmed. In the brain, the left hippocampus and its surroundings were moderately soft. The cerebral tissues of the limbic system showed spongiosis, neuronal loss, gliosis, mild perivascular cuffing of lymphocytes, and petechial hemorrhages (**Figures 1I, J**). There was no evidence of herpetic encephalitis, other infectious diseases, or prion disease. There was also no evidence of direct central nervous system invasion by the lymphoma, and no other tumor lesions were found throughout the body.

Immunohistochemistry on rat cerebrum using the avidin-biotin technique did not detect anti-neural antibodies, including anti-mGluR5 antibody and antibodies against other antigens (NMDAR, AMPAR, GABA_A_R, GABA_B_R, mGluR1, mGluR2, DPPX, IgLON5, Neurexin, LGI1, CASPR2) in serum and CSF obtained before steroid therapy. In previous studies (2–4), all samples were first screened for reactivity against rat brain neuropil by immunohistochemistry and cell-based assays (CBAs) for NMDAR. In the case of positive neuropil reactivity different from that of NMDAR antibodies, samples were investigated with CBA for antibodies to mGluR5 and other neuronal targets. Our case was examined by Prof. Josep Dalmau (Department of Neurology, University of Barcelona, Spain) and colleagues using the same process as in previous reports (2–4). Therefore, we can conclude that our case results are fully comparable with those of previous reports.

Although the antibodies were negative, the patient met all four of the diagnostic criteria (9) for autoimmune limbic encephalitis (Subacute onset, Bilateral abnormalities highly restricted to the medial temporal lobes on MRI, Electroencephalography with slow-wave activity involving the temporal lobes, and reasonable exclusion of alternative causes), and was diagnosed with definite autoimmune limbic encephalitis.

The diagnostic criteria formulated by the Paraneoplastic Neurological Syndromes Euro network (10) define limbic encephalitis as classical paraneoplastic neurological syndromes and include (i) subacute onset (days or up to 12 weeks) of seizures, short-term memory loss, confusion, and psychiatric symptoms; (ii) neuropathological or neuroradiological evidence of involvement of the limbic system; (iii) exclusion of other possible etiologies of limbic dysfunction; and (iv) demonstration of cancer within five years of the diagnosis of the neurological disorder or demonstration of a well-characterized paraneoplastic antibody. The presented patient fulfilled the criteria, and we diagnosed him as Ophelia syndrome (PLE with Hodgkin lymphoma).

## 3 Discussion

First, this patient had Ophelia syndrome with a negative anti-mGluR5 antibody. The presence of anti-mGluR5 antibodies has been confirmed in several cases of Ophelia syndrome (2–6). On the other hand, antibodies have been reported to be generally undetectable in most paraneoplastic syndromes associated with malignant lymphoma (8). However, recent reports of newly discovered antibody-mediated neurological diseases are reason enough to suspect the existence of many unknown antibodies, and we thought it worthwhile to report a case of PLE associated with Hodgkin lymphoma that was antibody negative. In addition, antibodies not included in most commercial panel centers, such as anti-mGluR5 antibodies, were difficult to measure in the other previous cases (11–15), and few institutions can evaluate the presence of antibodies. Therefore, to clarify the pathogenesis of the syndrome, it may also be necessary to know antibody-negative cases of Ophelia syndrome.

We reviewed all available published reports on Ophelia syndrome with anti-mGluR5 antibody testing, ascertained by a Pubmed search using the keywords: malignant lymphoma, limbic encephalitis, Ophelia syndrome, and mGluR5 (**Table 1**). All articles written in English which provided adequate clinical descriptions were reviewed. No case reports of anti-mGluR5 antibody-negative Ophelia syndrome were found, but one case was reported in Japanese, so we reviewed the case report. Although there were many cases in which the anti-mGluR5 antibody was not investigated, we did not include them because this study aims to compare cases according to the presence or absence of anti-mGluR5 antibody. As a result of the search, seven antibody-positive cases and one antibody-negative case were found. A summarized report of anti-mGluR5 antibody-positive limbic encephalitis (2) included 5 cases of Ophelia syndrome (PLE with Hodgkin lymphoma), and six other cases were associated with small cell lung cancer or had no tumor (17). Another case report describes an anti-mGluR5 antibody-positive encephalitis patient with worsening neurological symptoms after the initiation of chemotherapy for Hodgkin lymphoma, which improved with steroid pulse therapy (5). Comparing this present case with previously reported Ophelia syndrome positive for anti-mGluR5 antibodies (2–6), we found no significant differences in neurologic symptoms, including behavioral, personality, or mood changes, cognitive changes, decreased level of consciousness, or seizures. As in previous reports, our patients had mild prodromal symptoms, and neurologic symptoms preceded the tumor diagnosis. Head imaging and electroencephalographic findings were consistent with previous reports. All but one of the seven reported cases showed pleocytosis (2–6), while the CSF findings were almost normal in the antibody-negative cases, including the present case. Whether this difference is due to the presence or absence of an anti-mGluR5 antibody is unknown and should be evaluated after new case reports and case series of Ophelia syndrome are published.

**Table 1:** Clinical features of Ophelia syndrome with and without anti-mGluR5 antibodies.

Patient, Sex, age, y	Prodromal features	Tumor	Main clinical features; mRS score at peak of disease	CSF analysis	Increased T2/FLAIR signal on MRI	Treatment	Last follow-up, mo; outcome; mRS score	Antibody
Ophelia syndrome with mGluR5 antibodies								
F, 46 (2, 3)	None	HD, stage 3A	Personality changes and depression for 1 y, then seizures, memory loss, emotional lability, myoclonic jerks, and tremor. mRS score 3	23 WBC (Normal at first)	At onset: unilateral right mesiotemporal lobe. At 3 mo: bilateral temporal, thalamus, insula, frontal. Gd+	Steroids, ABVD	48; Complete recovery; mRS score 0	Serum: +; CSF: NA
M, 35 (2, 4)	Weight loss (9 kg)	HD, stage 2B	Aggressive behavior, depressed mood and anxiety, memory loss, right X, XI and XII nerve palsy. mRS score 3	12 WBC, no OCB, a raised IgG index	Bilateral (right > left) upper pons. Gd+	ABVD	38; Complete recovery; mRS score 0	Serum: NA; CSF: 1/160
M,16 (2)	Headache	HD, stage 3B	Psychosis, hallucinations, poor sleep, dystonia, generalized seizures, dLOC. mRS score 4. After complete recovery, neurologic relapse followed by tumor relapse	31 WBC, OCB	Normal	Steroids, chemotherapy, PE	48; Complete recovery; mRS score 0	Serum: >>1/1280; CSF: 1/20
M,15 (2, 3)	Headache, nausea	HD, stage 2A	Confusion, auditory and visual hallucinations, decreased verbal output, attention deficit, status epilepticus. mRS score 5	114 WBC, OCB	Bilateral (left > right) parietal-occipital cortical diffusion restriction	Chemotherapy, RT	72; Complete recovery; mRS score 0	Serum: NA; CSF: +++
M,15 (2)	None	HD, stage 1	Facial paralysis, then developed altered behavior, memory loss, anxiety, irritability, visual hallucinations, insomnia. mRS score 4	45 WBC, OCB	Normal	Steroids, IVIg, chemotherapy	12; Moderate memory problems; mRS score 2	Serum: 1/1280; CSF: 1/640
M, 68 (5)	Night sweats, low grade fever, weight loss	HD	Depression, anxiety, and memory loss. 6 mo later, disorientation, inattention, psychomotor agitation, confusion, delusional ideas of grandiosity, auditory hallucinations, and alterations of anterograde memory under the ongoing stable treatment with ABVD chemotherapy. mRS score NA	Cell count and proteins were normal, One OCB	A small established cerebellar infarction	ABVD, Steroids	1; complete recovery; mRS score NA	Serum: NA; CSF: +
F, 22 (6)	Headache	HD, stage 2	Right hemifacial allodynia, mild short-term memory disturbances and slight orientation problems in space and time. mRS score NA	Mild lymphocytic pleocytosis	Bilateral hippocampus and amygdala	ABVD, high-dose steroids, rituximab, PE	6; partial recovery; mRS score NA	Serum: +
Ophelia syndrome without mGluR5 antibodies								
M, 84 (16)	Unsteadiness, falls	HD, stage 3A	Depression, insomnia, memory loss. mRS score NA	Mildly elevated protein, OCB	Bilateral (right > left) thalamus	ABVD	6; complete recovery; mRS score NA	Serum: -, CSF: -
M, 72 (Our case)	Fatigue, anorexia, dyspnea	HD, stage 3A	Abnormal behavior, memory loss, dLOC, generalized seizures, myoclonic jerks. mRS score 6	Mildly elevated protein	Bilateral medial temporal lobes and left insular gyrus	Steroids	died	Serum: -, CSF: -

AVBD, chemotherapy with doxorubicin, vinblastine, bleomycin, and dacarbazine; dLOC, decreased level of consciousness; FLAIR, fluid-attenuated inversion recovery; Gd+, gadolinium enhancement; HD, Hodgkin disease; IgG, immunoglobulin G; IVIg, IV immunoglobulin; mGluR5, metabotropic glutamate receptor 5; mRS, modified Rankin Scale; NA, not available; OCB, CSF oligoclonal bands; PE, plasma exchange; RT, radiotherapy; WBC, white blood cells per mm3; +/+++, sample screened for reactivity against rat brain neuropil by immunohistochemistry and positive/strongly positive for mGluR5 cell-based assay; -, negative for mGluR5.

Excluded manuscripts are listed in **Supplementary 2**, and the reasons for their exclusion.

Second, we started steroids early, but this might have prevented the diagnosis of malignant lymphoma and the administering of chemotherapy. Prompt immunosuppressive therapy is recommended when we encounter patients who are suspected immune-mediated diseases, especially autoimmune encephalitis with severe neurological symptoms such as impaired consciousness and seizures. On the other hand, using steroids in malignant lymphomas may alter the histologic findings and complicate the biopsy itself, as in this case. Although PLE associated with malignant lymphoma is rare, this problem presents a significant dilemma for the clinician challenging a diagnosis of malignant lymphoma and associated common neurological disturbance.

PLE associated with Hodgkin lymphoma generally responds well to chemotherapy, and the prognosis is not poor (2). However, when the specific antibody is difficult to measure or negative, and biopsies cannot prove the presence of malignant lymphoma, we cannot start chemotherapy easily, and the patient’s prognosis can be disastrous. Therefore, if malignant lymphoma is strongly suspected, it may be necessary to attempt a biopsy as soon as possible with the cooperation of hematologists and surgeons. However, despite these efforts, the authors consider some cases where it is difficult to prove the association with malignant lymphoma and initiate chemotherapy. In such cases, even if there is a suspicion of Ophelia syndrome, the diagnosis is not definite, and many cases are not reported because autopsies have not been performed or for other reasons. We expect that the prognosis for such patients will improve as more and more cases, including suspected cases, become known.

This report has the following two limitations that need to be considered. Firstly, some tests, such as oligoclonal bands and IgG index, were not performed. However, there is sufficient evidence to suggest the existence of an autoimmune mechanism, as the patient displays clinical features that match the diagnostic criteria for autoimmune encephalitis (9). Secondly, while we used a fully comparable method to test anti-neuronal antibodies, the anti-mGluR5 antibody was not tested with a cell-based assay. A tissue-based assay has been shown to have the same sensitivity as the CBA method for anti-NMDAR antibodies (18), and we believe this is an alternative method.

## 4 Conclusion

We experienced Ophelia syndrome with a negative anti-mGluR5 antibody, and steroid therapy might have prevented the diagnosis of malignant lymphoma. We also believe it is essential to accumulate cases of this syndrome and clarify the association between PLE and Hodgkin lymphoma so chemotherapy can be initiated even if malignant lymphoma cannot be pathologically proven or when antibodies cannot be measured or are negative.

## Data availability statement

The original contributions presented in the study are included in the article/**Supplementary Material**. Further inquiries can be directed to the corresponding author.

## Ethics statement

The requirement of ethical approval was waived by Akita University Clinical Research Review Committee for the studies involving humans. The studies were conducted in accordance with the local legislation and institutional requirements. The participants provided their written informed consent to participate in this study. Written informed consent was obtained from the individual(s) for the publication of any potentially identifiable images or data included in this article.

## Author contributions

YS: conception, organization, writing, review, and critique; MM: pathological review and critique; HF, AH, SK, MS: review and critique. All authors contributed to the article and approved the submitted version.
